# Effect of aerobic exercise alone or combined with Mediterranean diet on dry eye in obese hypertensive elderly

**DOI:** 10.1007/s11845-023-03387-6

**Published:** 2023-05-09

**Authors:** Ali Mohamed Ali Ismail, Alshaymaa Shaaban Abd El-Azeim, Hatem Fawzy Abd Elfatah Abo Saif

**Affiliations:** 1 Department of Physical Therapy for Cardiovascular/Respiratory Disorder and Geriatrics, Faculty of Physical Therapy, Cairo University, Giza, Egypt; 2 Basic Science Department, Faculty of Physical Therapy, Cairo University, Giza, Egypt; 3 Department of Ophthalmology, Faculty of Medicine, Ain Shams University, Cairo, Egypt

**Keywords:** Diet, Dry eye, Elderly, Hypertension, Interval exercise, Obesity

## Abstract

**Background:**

Lifestyle modification is a newly recommended complementary treatment for dry eye (DE) disorder.

**Objective:**

To investigate the effect of a 6-month high-intensity interval aerobic exercise (HIIAE) (conducted 30 min, 3 times weekly) alone or combined with a caloric-restriction approach, the Mediterranean diet (MD), on DE parameters in obese hypertensive elderly.

**The design, settings, participants, and intervention:**

This is a randomized controlled trial included sixty obese hypertensive elderly with DE based on university-based hospital recruitment. Elderly were randomly assigned to the experimental group (*n* = 30 elderly received HIIAE plus MD) and control group (*n* = 30 elderly received only HIIAE). Besides anthropometry (abdominal circumference, body weight, and body mass index) and blood pressure (measured in systole and diastole), DE parameters (tear film break-up time, DE scoring system, ocular surface disability index questionnaire, Schirmer’s test, and Oxford grading system) were evaluated.

**Results:**

Significant improvements in anthropometry, blood pressure, and DE parameters were higher in the experimental group than in the control group.

**Conclusion:**

Aging-related DE symptoms and signs can be prevented and/or treated with HIIAE alone or combined with MD in obese hypertensive elderly with DE disorder.

## Introduction

Tear deficiency or excessive evaporation is the main descriptive definition of the tear film disorder, dry eye (DE). DE can damage the interpalpebral ocular surface with subsequent symptoms of ocular discomfort [1].

The symptomatic discomfort expresses itself in DE as ocular pain, itching, scleral redness, light sensitivity, fatigue or strain, heavy or foreign body sensation, and discharge [2]. Depression can be evoked in DE patients due to their poor quality of life (QoL). Daily activities dependent on vision are affected by DE disorder such as writing, reading, television watching, computer mobile use, and driving, so QoL is affected [3].

The global percentage of DE prevalence ranges from 5 to 87.5 [4]. Besides the older population, DE condition commonly affects women more than men, especially after menopause [5].

Physiological and structural ocular changes that occur with biological aging may explain the mechanism of DE in the elderly. Decreased tear secretion/volume, increased tear evaporation, apparent impairments of the lacrimal gland (histological, functional, and structural impairments), atrophy of the meibomian gland (this gland secretes the tear-containing lipid layer), and metaplasia of ocular orifices are local ocular changes predispose DE with aging. Low-grade chronic local and systemic inflammation is another apparent sign of DE in the elderly due to the aging of their immune system [4].

As life expectancy increases, the elderly tend to have a sedentary lifestyle. This lifestyle is the cause of elderly’s obesity and its associated diseases and/or complications including hypertension, diabetes, metabolic syndrome, and the highly prevalent DE [6]. The source of low-degree inflammation in obesity, adipose tissue, is rich in pro-inflammatory macrophages that produce pro-inflammatory markers. These markers exert some negative impacts on tear production [7].

Nowadays, the available medical treatments for DE are frustrating for sufferers and ophthalmologists [8]. Artificial tear treatments failed to replace or imitate normal tears [9]. Moreover, the topical treatments are uncomfortable to many patients [10], and the other popular medical treatment of DE, eye drops, encounters many problems. The commonly reported problems by patients are the cost of eye drops, failure to adhere to prescribed daily doses for a long time, partial cure of symptoms, not the disease [9], and the unpleasant instilling of eye drops many times per day [10]. Therefore, it is mandatory to find a new supporting complementary treatment for DE [9], especially with the new trend of incorporating complementary therapies in the treatment of other eye disorders [11–13].

Considering DE a lifestyle disease, the application of caloric restrictive and exercise approaches is an advisable lifestyle for DE patients [14]. Calorie restriction (CR) is proven to prevent or regress the aging-induced functional decline of different organs [15] including tear production in DE subjects [16]. Also, moderate-intensity exercise—applied in the continuous form—can produce the same effect of CR in healthy young subjects without DE [9]. To our limited knowledge, the efficacy of a 6-month high-intensity interval aerobic exercise (HIIAE) alone or combined with a caloric-restriction approach, the Mediterranean diet (MD), on DE variables in obese hypertensive elderly is not studied in DE literature, so this study was formulated.

## Materials and methods

### Study design

The design was as follows: single-blinded evaluation of DE parameters (conducted by an ophthalmologist who did not share in MD or HIIAE and both interventions were masked from him), the prospective registration of this lifestyle-modification study on clinicaltrials.gov (NCT05248698), and the randomized controlled followed designs.

### Setting

DE patients’ recruitment was managed from the ophthalmology outpatient clinic of Cairo University hospitals. The exercise sessions were supervised by the two physiotherapy authors of this study in a physical therapy center (a private center located in Cairo) from 5th January to 30th December 2022.

### Ethics considered during the study

Study conduction according to Helsinki recommendations, study and consent-form approvals from the local institutional ethical board (P.T.REC/012/003553; Faculty of Physical Therapy; Cairo University), consenting DE participants, and explanation of withdrawal rights to patients were the main ethical considerations of this study.

### Subjects

**Fig. 1:**
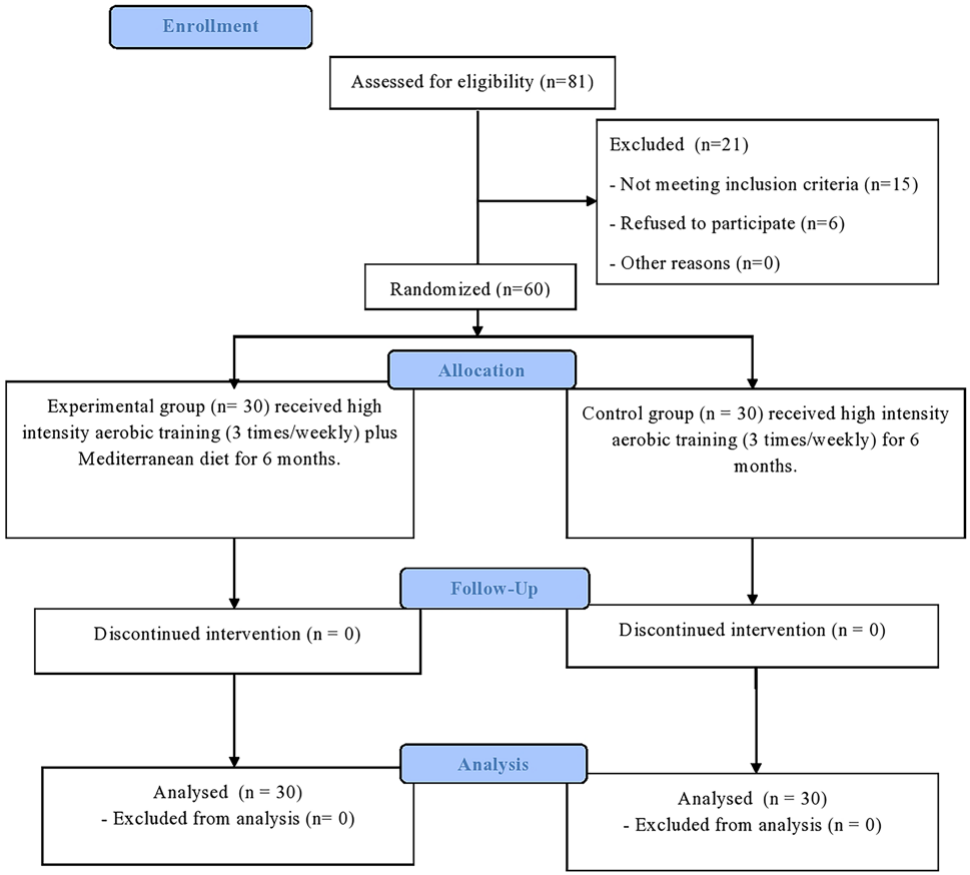
Dry eye interventional flow chart

The included elderly (≥ 65 years old) were randomly selected obese hypertensive patients with DE. The elderly who finished the assigned interventions were 60 patients (Fig. 1). The excluded criteria of elderly patients by the participating ophthalmologist author of this study were conjunctivitis, active infectious or allergic disorders, alcohol abuse habit, diabetes, autoimmune disorders, contact lens use, smoking habit, rheumatic diseases, previous surgeries of the eye (LASIK or punctal plugs), glaucoma, immunosuppressant disorders, local (facial nerve palsy) or general neurological disorders, deficiency of vitamin A, cardiorespiratory disorders, and any form of medical or complementary therapies for DE (oral supplementation of omega 3, topical ointments, eye drops, acupuncture, electro-stimulation, etc.) within the last 3 months. The two physiotherapy authors of this study excluded DE elderly who participated in diet or exercise programs within the last 6 months.

### Randomization protocol

The ED elderly were randomly and equally assigned via a computer-generated random list to the one of intervention-based groups (experimental and control groups). To guarantee the random blinded assignment of DE patients to groups, a physiotherapy practitioner (who did not know the interventions) planned the random list. For 6 months, both DE groups received HIIAE 3 times weekly while the experimental group received an additional MD plan.

### Interventions

#### High-intensity interval aerobic exercise

On an electronic bicycle, for an average of 30 min, every HIIAE was conducted. The exercise was initiated with an average 5-min warming-up phase (DE participant performed this phase at 60% of maximal heart rate, abbreviated as MHR). High-intensity interval aerobic bicycling (HIIAB) was allowed after the warm-up ended for 2 min (the 2-min HIIAB was performed at 90% of MHR and this was considered as one interval exercise phase). After the end of one interval exercise phase, the DE patient is allowed to rest in the form of an active moderate-intensity continuous bicycling phase (active rest phase) at 60% MHR for 1 min. The cycle of interval and active rest phases was repeated 7 times with an average time of 21 min. The exercise session ended with a cool-down phase which was similar to the criteria of warming up.

The elderly’s heart rate was monitored during every HIAB session using a heart rate monitor that was worn at the elderly’s wrist during bicycling.

#### Protocol of MD

The MD protocol of this study was based on the recommendations of Bedard et al. [17]. The weekly consumption of virgin olive oil was 320 ml. The daily consumption of cereals or bread was 30 g. Whole grain products (couscous, Egyptian rice, pasta, and bulgur), fruits, and vegetables shared the same serving-size consumption per day, 125 ml for each. Eggs were consumed in a 100-g serving size daily. The serving size for legumes and nuts was 175 ml and 30 g respectively. Patients who were urged to eat red meat, they allowed eating 75 g serving size weekly which was equal to the serving size of fish and poultry which was taken daily. One-time consumption of low-fat dairy products was allowed weekly (250 ml milk, 50-g cheese, and 175-g yogurt).

As a result of the above-designed MD protocol, the meal plan consisted of around 50% carbohydrates (the main source for carbohydrates was legumes, vegetables, fruits, and whole-product grains), 35% fats (the main source for fats was healthy oils, nuts/seeds, and fish), and 15% proteins (the main source for proteins was nuts, egg, poultry, low-fat dairy products, legumes, and fish).

Based on the total daily requirement and degree of DE-participant’s activity (2000 kcal/d for ♂ and 1600 kcal/d for ♀), a calorie restriction of about 15% from the daily Kilocalories was proposed.

During the 6-month research period, weekly consultations with a nutritionist were used to give nutritional counseling. The weekly visit was a chance for the nutritionist to assess patients ' diet adherence. All DE participants were taught how to complete a 24-h recall dietary evaluation of consumed foods, which was examined at each face-to-face nutritional session with the nutritionist.

#### Prerequisite assessment (cardiopulmonary exercise test)

This test was conducted one time only (before the conduction of the first HIIAB session in both study DE groups on an ergometer cycle). In order to make the patients adapt to the test steps during the test’s beginning, unloaded cycling was maintained for 3 min. To incorporate the patients in warming up, the cycling was loaded by 40 Watt. After the termination of the warm-up that reached 3 min, the examiner started to increase the load on the patient to 20 Watts every 60 s. The aim of this incremental loading was to reach the DE patients to one of the two following notices: patients’ sensed fatigue or 90% of MHR. If one notice was achieved, cooling down was started with the same criteria of warming up. The test was finalized with the unloaded cycling (0 watts for 3 min) [18, 19].

#### Pre and post-assessments

##### Blood pressure and anthropometry

Diastolic blood pressure (DBP), systolic blood pressure (SBP), weight, body mass index (BMI), and waist circumference (WC) were measured in all DE patients.

##### DE scoring system (DESS)

To assess the symptoms related to DE, this questionnaire was used. DESS asked about the presence of 6 items: eye fatigue, ocular itching/burning, local redness, gritty/sandy sensation, blurred vision, and abnormal excessive blinking. Every item is represented with an answer of zero (absent), one (sometimes), two (frequent), and 3 (always found). The score of DESS ranged from zero (minimal score) to 18 (maximal severe score) [20].

##### Ocular surface disability index (OSDI questionnaire)

It is a valid surOcular surface disability index (OSDI questionnaire)vey that rates the severity of DE patients’ environmental-activated ocular symptoms and complaints in the previous week. The survey consists of 12 items, each of which is evaluated on a scale of 0 to 4, with 0 being an indicator of none of the time and 4 being an indicator of all of the time. The OSDI questionnaire includes a total score as well as 3 subscale scores: vision-associated symptoms (5 items), ocular symptoms (4 items), and environmental triggers or stimuli (3 items). The overall score of the OSDI questionnaire is gained by dividing the total score of all answered questions by the number of answered questions and then multiplying the result by 25. Then, the result of this equation was assessed on a 0–100 scale with higher scores detecting the ocular impairment. A 0–12 score of OSDI is an indicator of a normal eye (absence of DE). An OSDI score ≥ 13 is a diagnosis of dry eye [21].

##### Schirmer’s test

This test was done to assess the speed of tear production. After 2 min of using topical anesthetic eye drops (0.4% benoxinate hydrochloride; Benox; Eipico, made in Egypt), the test was initiated. The test was conducted by the placement of Whatman’s no. 41 filter paper strips (5-mm width × 35-mm length) in the inferior conjunctival fornix (the filter paper was placed at the junction of the lateral $^{1}/_{3}$ and medial $^{2}/_{3}$ of inferior fornix). The patient was ordered to continue blinking without closing his/her eyes and after 5 min, the strips were removed from both eyes by the examiner. The strip’s length wetted by tears was measured. For the statistical analysis, the mean Schirmer test scores of the right and left eyes were employed [22]. Score < 10 mm was considered a borderline DE [23].

## Tear film break-up time (TBUT) testing

It was done to assess tear film stability (i.e., time needed for tears to be evaporated and diffused after natural blinking). Briefly, sodium fluorescein (1% eye drops; Minims Fluorescein sodium; Baush + Lomb, Kingston-upon-Thames, England) was dropped into the conjunctiva sac. The participants were ordered to blink 3–4 times and then look forward without any blinking. The tear film was examined under the slit-lamp biomicroscope light. The time between the last blink and the emergence of the first break in the tear film (i.e., formation of a dry spot on the cornea) was recorded using a timer [24]. The mean TBUT was compu+ted after the measurements were accurately done three times. Every eye received the same TBUT assessment. The statistical analysis was conducted using the mean TBUT scores of the right and left eyes [22]. DE has considered if the TBUT value was less than 5 s [22, 24].

## Oxford grading system

In DE sufferers, the Oxford grading system (OGS) was created to determine the extent of epithelial surface damage of conjunctiva and cornea damage. To initiate OGS, instilling a 10-µl fluorescein sodium in the superior conjunctiva was required. The OGS method employs a chart comprised of a sequence of panels (figures) referenced from A to E in ascending degree of severity. Staining is shown by punctate dots in each figure. From panels A to E, the number of drawn dots within every panel increases [25]. The examiner analyses the patient’s corneal staining’s overall look and then matched it with its corresponding within-chart figure that best describes the degree of the patient’s corneal staining [26].

The pre-interventional selected criteria for DE diagnosis in our study participants were based on the presence of at least one reported DE symptom on DESS [27] and OSDI score ≥ 13 [21] combined with either TBUT value < 5 s [22, 24] or less than 10-mm-score Schirmer’s test [23].

### Sample size calculation

The number of DE patients was calculated by the popular estimating sample size program; G*power. This calculation was postulated in a conducted pilot study by the manuscript’s authors on 10 DE patients. The result for this calculation marked the sample size = 54 DE patients considering Schirmer’s test was the primary outcome. The value of 0.68 was the resultant effect size which was estimated on the statistical program with type II error = 0.8 and type I error = 0.05. Then, the number increased by 10% due to the estimation of dropout. So the final needed DE elderly were 30 patients in every group.

### Statistical analysis

Shapiro-Wilk test was used for investigating the normality of age, blood pressure anthropometry variables (height, weight, BMI, and abdominal circumference), duration of DE symptoms, and DE variables (DESS, OSDI questionnaire, TBUT, and Schirmer’s test). All previously mentioned variables were normally distributed, so parametric tests were used for analysis.

Because of the non-normal distribution, a non-parametric test was used for OGS analysis. An unpaired *T*-test was used to elucidate the difference between both-group physical characteristics and baseline measures of all dependent variables except OGS. For within-and between-group OGS analysis, Wilcoxon signed rank test and Mann–Whitney test were used.

Two-way MANOVA was employed to illustrate the difference between time (pre and post) and treatments (DE groups). *F*-value determination was managed through Wilks’ lambda test. The post-hoc test was performed to analyze the difference between pre and post within each DE group, as well as to find differences between DE groups at pre-treatment and post-treatment variables.

To measure the size of the difference between DE groups, a partial eta square (*η*^2^) was used. Regarding the sex factor, the chi-square (*X*^2^) test was performed to differentiate across DE groups. SPSS (version/23) (New York, USA) was used, and *α* = 0.05 was applied.

## Results

Figure 1 demonstrates the flow chart of this DE study. Eighty-one hypertensive obese elderly with DE were recruited. Twenty elderly were uninvolved due to mismatches with the authors’ inclusion criteria. Fifteen DE elderly declined to participate in this lifestyle-modification study, and six DE elderly had a busy schedule. So, sixty elderly (one hundred and twenty DE complaints) were included in the analysis, and they were divided into two equal elderly groups at random way. The physical characteristics of the elderly and their baseline measures were shown in Table 1 which shows no significant difference between both elderly groups as *p*-value < 0.05.

**Table 1:** Demographic data and baseline measurements of all dependent variables in this dry eye study

		**Mean ± SD**		***p*-value**
		Experimental group	Control group	
**Age: (years)**		66.06 ± 4.42	65.96 ± 3.88	0.92^**^
**Weight**: **(kg**)		96.4 ± 5.75	97.26 ± 6.37	0.58^**^
**Height**: **(cm**)		170.23 ± 5.59	169.13 ± 6.43	0.48^**^
**BMI: (kg/m**^**2**^**)**		33.33 ± 1.66	34 ± 2.1	0.17^**^
**Duration of symptoms (m)**		36.43 ± 4.11	37.76 ± 3.55	0.18^**^
**Abdominal circumference (cm)**		114.03 ± 7.26	115.93 ± 6.41	0.28^**^
**Systolic blood pressure (mm Hg)**		167.4 ± 3.67	168.4 ± 2.66	0.23^**^
**Diastolic blood pressure (mm Hg)**		98.6 ± 2.47	97.7 ± 2.49	0.16^**^
**Gender (male/female)**		10 males/20 females	12 males/18 females	(*X*^2^ = 0.28) *p* = 0.59^**^
**Eye parameters**				
** DESS**	***Right***	4.31 ± 0.53	4.51 ± 0.5	0.14^**^
	***Left***	4.35 ± 0.54	4.45 ± 0.54	0.49^**^
** OSDI questionnaire**	***Right***	41.16 ± 4.71	42.3 ± 5.17	0.37^**^
	***Left***	41.46 ± 4.73	41.8 ± 5	0.79^**^
** TBUT(sec)**	***Right***	5.29 ± 0.48	5.18 ± 0.39	0.31^**^
	***Left***	5.32 ± 0.48	5.31 ± 0.4	0.36^**^
** Schirmer’s test (mm)**	***Right***	3.33 ± 0.52	3.2 ± 0.45	0.28^**^
	***Left***	3.3 ± 0.51	3.15 ± 0.49	0.26^**^
** Oxford grading system**	***Right***	0.63 ± 0.08	0.61 ± 0.07	0.3^**^
	***Left***	0.62 ± 0.07	0.6 ± 0.083	0.22^**^

*SD* standard deviation, *p*-value significance level, *BMI* body mass index, *X*^2^ chi-square test, *cm* centimeter, *m* month, *mm Hg* millimeter of mercury, *DESS* Dry Eye Scoring System, *mm* millimeters, *OSDI* Ocular Surface Disease Index, *sec* seconds, *TBUT* tear break-up time

^**^No significance difference

According to MANOVA analysis in the participated elderly with DE, there were statistical significant impact at the studied elderly groups as Wilks’ Lambda (*ʎ*) = 0.42, *f* = 6.73 and *p* = 0.001, and* η*^2^ = 0.58; also, there was a statistically significant impact in time (pre and post-assigned lifestyle treatments) as *ʎ* = 0.02, *f* = 213.97, *p* = 0.0001 and *η*^2^ = 0.97; finally, there was a statistically significant impact at the interaction between time and the two studied elderly groups as *ʎ* = 0.14, *f* = 29.95, *p* = 0.0001 and *η*^2^ = 0.85.

### Within and between elderly groups’ analysis

Tukey-test pairwise comparisons between pre-and post-treatment measures within DE groups reported a statistically significant decrease in weight in both DE groups as the mean difference (Md) in the experimental group (HIIAE plus MD) was 10.03 and in the control group (HIIAE) was 5.33 so, the superiority of treatment for the group of HIIAE plus MD. In BMI, also, there were a statistically significant decrease as Md = 3.46 and 1.9 in the experimental (HIIAE plus MD) and control group (HIIAE), respectively. The abdominal circumference also decreased as Md = 10.13 and 6.3 in the experimental (HIIAE plus MD) and control group (HIIAE), respectively, with more favor to the experimental (HIIAE plus MD) group. Finally, blood pressure in systole and diastole had a significant decrease in both DE groups as MD = 6.53 and 4.13 in systole and 8.03 and 3.8 in diastolic blood pressure with more difference in the experimental group (HIIAE plus MD). At post-treatments, regarding weight, BMI, abdominal circumference, and blood pressure, the between-DE group analysis reported a statistically significant difference between both DE groups (*p* < 0.05) (Table 2).

**Table 2:** Within and between group analysis of weight, body mass index, abdominal circumference, and blood pressure

**Measured variables**	**Experimental group**	**Control group**	***p***-**value between groups**	***F***-**value between groups**	***η***^2^
**Weight (kg)**					
Post-treatment	86.36 ± 4.2	91.93 ± 5.71	0.0001^*^	18.48	**0.24**
* p*-value (within)	0.0001^*^	0.001^*^			
MD	10.03	5.33			
95% CI	8.54 to 11.52	3.84 to 6.82			
**Body mass index (kg/m**^**2**^**)**					
Post-treatment	29.86 ± 1.85	32.1 ± 2.04	0.001^*^	19.71	**0.25**
* p*-value	0.001^*^	0.003^*^			
MD	3.46	1.9			
95% CI	2.94 to 3.99	1.37 to 2.42			
**Abdominal circumference (cm)**					
Post-treatment	103.9 ± 5.6	109.63 ± 6.37	0.0001^*^	13.69	**0.19**
* p*-value (within-group)	0.0001^*^	0.001^*^			
MD	10.13	6.3			
95% CI	8.74 to 11.52	4.91 to 7.68			
**Systolic blood pressure (mm Hg)**					
Post-treatment	160.86 ± 3.28	164.26 ± 3.48	0.0001^*^	15.11	**0.21**
* p*-value	0.001^*^	0.001^*^			
MD	6.53	4.13			
95% CI	5.67 to 7.39	3.27 to 4.99			
**Diastolic blood pressure (mm Hg)**					
Post-treatment	90.56 ± 3.5	93.9 ± 1.44	0.0001^*^	23.23	**0.28**
* p*-value	0.001^*^	0.001^*^			
MD	8.03	3.8			
95% CI	6.99 to 9.07	2.75 to 4.84			

*P* value less than 0.05

*SD* standard deviation, *p*-*value* significance level, *MD* mean difference, *cm* centimeter, *mm Hg* millimeter of mercury

^*^Significant difference

### Within and between-group analyses of the elderly’s both eye parameters

According to the analysis of both eyes’ parameters, there were improvements in DESS and OSDI questionnaires as the value decreased after treatment with more favor to the experimental group (the group received HIIAE plus MD). Also, TBUT and Schirmer’s test increased after the treatment in patients’ right and left eyes in experimental and control groups with more superiority to the experimental group. Finally, OGS showed a significant decrease in both groups as MD = 0.04 and 0.01 (right eye) and 0.05 and 0.01 (left eye) in the experimental and control group respectively. Between-group analysis at post-treatment there was a significant difference between both DE groups at all variables, except for OGS as *p* = 0.53 and 0.69 in right and left eye, respectively (Tables 3 and 4).

**Table 3:** Within and between group analysis of right eye parameters

**Measured variables**	**Experimental group**	**Control group**	***p*-value between groups**	***F*-value between groups**	***η***^**2**^
**DESS**					
Post-treatment	3.5 ± 0.59	4.21 ± 0.5	0.0001^*^	24.99	**0.3**
* p*-value (within)	0.0001^*^	0.006^*^			
MD	0.81	0.3			
95% CI	0.6 to 1.02	0.09 to 0.51			
**OSDI questionnaire**					
Post-treatment	35.56 ± 4.62	38.1 ± 4.16	0.03^*^	4.96	**0.08**
* p*-value	0.0001^*^	0.001^*^			
MD	5.6	4.2			
95% CI	4.51 to 6.68	3.11 to 5.28			
**TBUT**					
Post-treatment	6.73 ± 0.68	5.76 ± 0.45	0.0001^*^	41.23	**0.41**
* p*-value (within-group)	0.0001^*^	0.001^*^			
MD	− 1.43	− 0.58			
95% CI	− 1.6 to − 1.27	− 0.75 to − 0.42			
**Schirmer’s test (mm)**					
Post-treatment	4.72 ± 0.7	3.76 ± 0.36	0.0001^*^	43.9	**0.43**
* p*-value	0.0001^*^	0.0001^*^			
MD	− 1.38	− 0.56			
95% CI	− 1.55 to − 1.21	− 0.73 to − 0.39			
**Oxford grading system**					
Post-treatment	0.58 ± 0.08	0.59 ± 0.07	0.53^**^	*Z* = − 0.96	**0.007**
* p*-value	0.001^*^	0.001^*^			
MD	0.04	0.01			
95% CI	0.006 to 0.09	0.02 to 0.05			

*P* value less than 0.05

*SD* standard deviation, *p*-value significance level, *MD* mean difference, *DESS* Dry Eye Scoring System, *mm* millimeters, *OSDI* Ocular Surface Disease Index, *sec* seconds, *TBUT *tear break-up time

^*^Significant difference; ^**^non-significant difference

**Table 4:** Within and between group analysis of left eye parameters

**Measured variables**	**Experimental groups**	**Control groups**	***p-*value between groups**	***F*-value between groups**	***η***^**2**^
**DESS**					
Post-treatment	3.41 ± 0.58	4.17 ± 0.59	0.0001^*^	24.91	**0.29**
* p*-value (within)	0.0001^*^	0.015^*^			
MD	0.94	0.28			
95% CI	0.72 to 1.17	0.05 to 0.51			
**OSDI questionnaire**					
Post-treatment	35.06 ± 4.22	38.53 ± 4	0.002^*^	10.5	**0.15**
* p*-value	0.0001^*^	0.0001^*^			
MD	6.4	3.3			
95% CI	5.05 to 7.74	1.91 to 4.61			
**TBUT**					
Post-treatment	6.79 ± 0.74	5.66 ± 0.44	0.0001^*^	51.48	**0.47**
* p*-value (within-group)	0.0001^*^	0.0001^*^			
MD	− 1.47	− 0.447			
95% CI	− 1.69 to − 1.24	− 0.66 to − 0.22			
**Schirmer’s test (mm)**					
Post-treatment	4.79 ± 0.72	3.81 ± 0.45	0.0001^*^	39.29	**0.4**
* p*-value	0.0001^*^	0.0001^*^			
MD	− 1.48	− 0.66			
95% CI	− 1.7 to − 1.26	− 0.88 to − 0.44			
**Oxford grading system**					
Post-treatment	0.56 ± 0.08	0.58 ± 0.08	0.69^**^	*Z* = − 0.39	**0.006**
* p*-value	0.046^*^	0.049^*^			
MD	0.05	0.01			
95% CI	0.012 to 0.04	0.03 to 0.06			

*P* value less than 0.05

*SD* standard deviation, *p*-*value* significance level, *MD* mean difference, *DESS* Dry Eye Scoring System, *mm* millimeters, *OSDI* Ocular Surface Disease Index, *sec* seconds, *TBUT* tear break-up time

^*^Significant difference; ^**^non-significant difference

## Discussion

This study proved that exercise (HIIAB) can significantly relieve DE symptoms. DE relief can be also maximized by the additional effect of MD in obese hypertensive elderly with DE. The mechanism that explains the effect of MD or exercises on DE is not fully sufficient in the literature.

The popularity of MD in improving cardiovascular risk factors (including obesity) [28] and general health of body organs including the eye [10] is very wide. This may return to the consumed dietary items of MD protocols [28].

The included olive oil, vegetables, poultry, nuts, cereal products, and sometimes meat are main sources of some essential fatty acids (EFAs) as omega-6 EFAs (O6EFAs). The included marine oil and fish are sources of other EFAs, omega-3 EFAs (O3EFAs) [29]. Despite the inability of the normal human body to synthesize O3EFAs and O6EFAs, dietary consumption of both EFAs is very important to general health [30].

It is supposed that the good choice to within-diet healthy foods maintains the ratio of O6EFAs: O3EFAs within 1:1. This ratio can maintain regular inflammatory and immune responses within the human body. Some unhealthy Western diets are low in O3EFAs, so the ratio become 17:1 [30].

The high level of O6EFAs accelerates the production of pro-inflammatory mediators including PGE2 and LTB4. The low level of O3EFAs cannot block the formation of interleukins and tumor necrosis factor-alpha. A higher O3EFAs to O6EFAs ratio exerts an anti-inflammatory and immune-enhancing effect. The health advantages of dietary O3EFAs consumption have been documented for DE [20]. Considering DE is a chronic inflammatory process initiates with the tear-hyperosmolarity stage to be promoted in ocular changes that manifest the symptoms and severity of DE [31]. According to these considerations, recently, O3EFAs and O6EFAs oral supplementation are supposed to lower the expression of conjunctival pro-inflammatory markers [32], improve inflammatory ocular-surface processes, and relieve DE symptoms [33].

As an alternative to oral supplementation, the integrative components of MD foods contain the needed O3EFAs and O6EFAs for the maintenance of healthy connective tissue [29] including the ocular tissues. The O6EFAs: O3EFAs ratio may be regulated with long-term adherence (6 months) on a dietary pattern as MD. The good adherence of DE participants to our MD protocol may be the leading cause of improved immune system, reduced ocular tissue damage, and lowered obesity- and aging-induced processes (these processes are usually responsible for the massive oxidative damage and chronic low-grade systemic inflammation that commonly occur in DE).

As it was previously proven in immortalized epithelial cells of the human meibomian gland, the regular administration of O3EFAs and O6EFAs can improve the quality and quantity of intracellular lipids that maintain tear production from the lacrimal gland [34].

Virgin olive oil, as an MD component, contains a large oleic-acid (monounsaturated fatty acid) content. This fatty acid protects the organs’ tissue against oxidative damage. Also, hydroxytyrosol, as a phenolic compound present in virgin olive oil, has anti-oxidative, anti-inflammatory, and neuroprotective properties. These properties may exert their effects on the health of the cardiovascular system [35] and ocular tissue.

Regarding exercise, the exercise-induced harmony between the sympathetic and parasympathetic nervous systems may be the cause of increased tear production. This regulated harmony can stimulate the within-eye epithelial sodium channels. This stimulation, in turn, can increase sodium reabsorption which plays a vital role in the maintenance of water and electrolytes found in produced tears [9].

As it was evidence-based in the literature, exercise-induced reduction of adipose tissue improves the associated cascades of chronic low-grade systemic inflammation present in cardiovascular risk factors [36]. Consequently, declined expression of inflammatory cytokines found in the tears of DE patients may justify increased tear production after exercise in humans [9].

Despite the low number of published studies, a recent study supported our results because its authors documented a decrease in tear production (assessed by Schirmer’s test) in systemic hypertension patients when matched with normal control subjects [37]. Supporting the presence of DE in hypertensive patients that is reported in our study, recent studies documented a high DE prevalence reached 20% [5] and 48% [6] in systemic hypertension patients.

The results of a relatively new published animal study supported our results. This animal study stated two facts. The first fact was the low rate of tear production in obese mice. The second one was the ability of exercise to increase this rate in addition to the suppression of reactive oxygen specimens (ROS) found in the lacrimal gland of this obese mouse [38]. This finding supports the involvement of high ROS rates [39] and low antioxidant levels in DE pathogenesis [40].

Another study conducted on rats in 2010 showed that CR is a new therapeutic tool that can be utilized to prevent age-associated dryness of the eye or lacrimal gland dysfunction. This animal study concluded that there is a positive role of a 6-month CR approach in the significant improvement of tear volume, tear protein, oxidative-stress-related destruction in the lacrimal gland, and preservation of lacrimal gland functions and structures (decreased interstitial fibrosis, preserved mitochondrial structure, and increased density of acinar unit of lacrimal gland) [15].

The published studies assessing the effects of lifestyle changes on human DE are very low. Supporting our opinion, weight loss through 6-month lifestyle changes (MD alone or combined with 45-min walking/day) can cause a significant improvement of TBUT, OGS, DESS, Schirmer’s test, and OSDI questionnaire in DE participants with metabolic syndrome [16].

Supporting the role of exercise in preventing DE, another study showed a significant increase in tear production and a decrease of tear cytokines after involving 43 without-DE healthy participants aged = 23.3 ± 3.6 years in one session of moderate intensity 0.5-h aerobic exercise on a treadmill [9].

Also, another study reported an association between DE and sedentary behavior or lower level of energy expenditure in humans [41]. Increased sedentary behavior may cause metabolic changes, and tear output may deteriorate over time as a result [42]. As supporting documentation to our results, another study reported a significant improvement of subjective symptoms after involving eleven DE-symptom office workers (aged 31 to 64 years old) in a 10-week energy-expenditure exercise program [42].

Despite the observed improvement of subjective symptoms and the number of definite criteria diagnosing DE, the results of objective tests (staining score, TBUT, and Schirmer’s test) conducted by Kawashima et al. [43] opposed our findings. This occurred because of the non-significant improvement of these objective tests after including 17 middle-aged office workers with definite or anticipated DE in a short-duration 2-month lifestyle-change program (diet modification, energy-expenditure increase through exercise, and positive thinking encouragement).

## Limitations

The authors of this paper did not track results beyond the only measurement they did after the end of the six months of treatment, and therefore it is required to examine this part in the future.

Future studies are requested to examine DE’s response to MD versus other weight-loss diets, HIIAB versus moderate-intensity energy-expenditure exercises, and aerobic versus resistance exercises.

## Conclusion

In light of the results presented in this research, DE patients who maintained a 6-month energy-expenditure protocol (a 30-min high-intensity aerobic bicycling, 3 times per week) exhibited more significant improvements in subjective and objective DE parameters when this exercise protocol accompanied by a dietary restriction program, Mediterranean diet.

## Data Availability

The data of this dry eye paper will be available on request.
